# Characteristics in patients with headache in an outpatient clinic in Japan

**DOI:** 10.1186/1447-056X-9-10

**Published:** 2010-11-18

**Authors:** Toshikatsu Okumura, Sachie Tanno, Masumi Ohhira, Satoshi Tanno, Tsukasa Nozu

**Affiliations:** 1 Department of General Medicine, Asahikawa Medical University, Asahikawa, Japan

## Abstract

**Background:**

Little is known about the prevalence of primary and secondary headache in clinics in Japan. The aim of this study is to characterize patients with headache in an outpatient unit where primary care physicians are working in Japan.

**Methods:**

Consecutive outpatients who newly visited the Department of General Medicine, Asahikawa Medical College Hospital, Asahikawa, Japan between April 2005 and March 2009 were analyzed. Each parameter such as age, sex or diagnosis was investigated.

**Results:**

Out of 4693 patients, 418 patients visited to our department because of headache. Primary headache was found in 167 patients (39.9%). The rate of tension-type headache (TTH) (30.8%) was highest, followed by migraine (9.1%). Approximately 3 times higher rate of migraine was observed in female patients when compared with male patients. In female patients, migraine was observed more frequently in younger patients. On the other hands, TTH was observed in almost all aged patients in males and females, and the rate of TTH peaks between the ages of 40 and 49 years in both sex. The present study also demonstrated that 8.4% of patients who chiefly complained of headache had been diagnosed as depression while 1.7% of remained patients had been diagnosed as depression, indicating 5-times higher rate of depression in patients with headache.

**Conclusion:**

All these results suggest that primary headache, especially TTH, is highly observed and depression should be considered in patients with headache in an outpatient clinic where primary care physicians are working in Japan.

## Background

Headache is the most prevalent neurological symptom and is experienced by almost everyone [1]. Population-based epidemiological studies on primary headache have been carried out in many countries [2]. Stovner et al. have described in the review article that there exists a regional differences in the prevalence of primary headache [2]. For example, the prevalence of tension type headache (TTH) in the adults in Europe is much higher than other regions including North America and Asia, and the prevalence of migraine in Asia is lower than Europe and North America. Although there are two reports on the prevalence of primary headache such as migraine in general population in Japan [3,4], little is known about the prevalence of not only primary headache such as migraine and TTH but also secondary headache in outpatient setting where primary care physicians are working in Japan. The aim of this study is therefore to characterize patients with headache in Japan who seek medical treatment.

## Methods

We analyzed consecutive outpatients who newly visited at Department of General Medicine, Asahikawa Medical College Hospital. In Japan, almost everyone is covered by national health insurance. Patients generally have the freedom to choose the health care provider that feel best fits their needs without concerns regarding costs. Therefore, there might be no big difference between patients who visit the department of General Medicine in Asahikawa Medical College hospital and ones who visit clinics or smaller hospitals. The total number of patients during April 2005 and March 2009 evaluated in this study was 4693. As reported previously [5,6], Asahikawa Medical College Hospital is located in Asahikawa City which has a population of approximately 350,000 in the middle of the Hokkaido Island, the most northern part of Japan. The hospital consists of 602 beds in which approximately 250 doctors are working to cover almost all of medical problems. Among them, 5 or 6 primary care physicians are working at the Department of General Medicine. New patients who come to the hospital by an ambulance are always admitted to the emergency unit in this hospital. Therefore, such patients are not admitted in the department of General Medicine. Out of new walk-in patients who visited to outpatient departments in our hospital, the patients who have a letter from other doctors to a specific department such as the department of Dermatology go to directly to the department. On the other hand, patients who have no letter from other doctors and do not know "I should visit to what department" come first to the Department of General Medicine. In our recent paper [5], evaluated outpatients who visited the Department of General Medicine were classified into the major ICD-10. Thus a large variety of patients visited to the Department of Medicine, Asahikawa Medical College Hospital, Japan.

All data were drawn from medical records and Computerized Physician Order Entry System in the hospital. Each parameter such as age, sex and diagnosis was investigated from the source. All patients who accepted for examination of laboratory test, we measured routinely urine and blood samples. These include complete blood count, liver function, renal function, C-reactive protein and TSH. To exclude the secondary headache, patients who complained of symptoms such as headache with increasing intensity, sudden onset and/or headache that had been never experienced before, were received brain CT scan to rule out brain diseases. Rapid test for influenza virus, blood test for detection of EB virus or culture tests that had been considered to be necessarily for differential diagnosis was performed in patients who had obvious signs of acute infection, like fever of ≥ 37.0°C within a couple of days. The International Classification of Headache Disorders version 2 (ICDH-II) [7] was applied to each patient who complained of headache.

Depression and other psychiatric disorders were diagnosed according to DSM-IV [8]. Due to the clinical limitation, DSM-IV classification did not apply for all patients. For instance, patients with acute fever, abdominal pain and watery diarrhea were diagnosed as acute enterocolitis without DSM-IV classification. As one may speculate, we could not exclude the possibility if the patient might be depression then. Thus, there was a clinical limitation when the patients visited to our clinic. In fact, DSM-IV classification was applied to patients who complained headache chronically. On the other hands, it is certain that a part of patients who complained symptoms other than headache were diagnosed as depression. When we wonder if the patient may be depression because we observed appetite loss, sleep disturbance, unexplained body weight loss, recent suicide attempt, our feeling "He/She looks depressive" and so on, final diagnosis of depression have been done according to DSM-IV. Statistical analysis was made using χ square test using IBM SPSS statistics software for Windows version 18.0 (SPSS Inc., Chicago, USA). A level of p < 0.05 was considered to be statistically significant.

## Results

Out of 4693 patients, 418 (8.9%) patients visited because of headache. CT scan or MRI imaging to detect or exclude brain disorders was performed in 85 patients (20. 3%) out of the 418 patients. Final diagnosis of 418 patients was shown in Table 1. The present results demonstrated that cerebrovascular disorders had been rarely observed in patients who complained of headache as shown in Table 1. Primary headache was found in 167 patients (39.9%). The rate of TTH was highest, followed by migraine. Secondary headache was found as in Table 1. These include systemic infection such as influenza and common colds, rhino sinusitis or depression. Psychiatric disorders other than depression include schizophrenia and, neurotic and somatoform disorders. When systemic infection is excluded, the rate of primary headache such as TTH and migraine out of all patients with headache is 49.4% (167/338), and the prevalence of primary headaches and depression is approximately 60.0% (202/338).

**Table 1:** Diagnosis of patients who had headache

ICHD-II diagnosis		Number	(%)
Migraine		38	9.1
Tension-type headache		129	30.8
			
cerebrovascular disorder		3	0.7
non-vascular intracranial disorder		2	0.5
systemic infection (bacterial or viral)		80	19.1
disorder of homoeostasis	thyroid	3	0.7
	hypertension	5	1.2
eyes, ears, nose, sinuses, structures	acute glaucoma	1	0.2
	rhinosinusitis	11	2.6
psychiatric disorder	depression	35	8.4
	others	29	6.9
Cranial neuralgias and central causes of facial pain	herpes zoster	7	1.7
			
Others		75	17.9
			

Total		418	

Table 2 summarized the data on the gender and age distribution of migraine, TTH and depression who complained of headache in the present study. Based on the Table 2, the rate of migraine and TTH in all patients was 0.8 or 2.9%, respectively, indicating TTH is the highest prevalence in this study. With regard to the relationship between sex or age, and the number of patients with migraine and TTH, there was a significant gender difference in the frequency of migraine in all aged patients (male vs. female, 0.4% vs 1.1%), indicating a higher rate of migraine in female patients. It was also shown that migraine was limited to patients aged 20-39 years old in male patients. In female patients, migraine was observed more frequently in patients aged 20-39 years old. There were significant numbers of migraine older than 40 years in women but not in men. On the other hands, the rate of TTH was 1.6 times higher in female than male. Although TTH was observed in almost all aged patients in males and females, the rate of TTH peaks between the ages of 40 and 49 years in men and women.

**Table 2:** Gender and age distribution of migraine, tension type headache and depression

	Age	Total patient	Tension type		
			Migraine	Headache	Depression
**Male**					
	10-19	112	0	0	0
	20-29	331	4 (1.2%)	6 (1.8%)	3 (0.9%)
	30-39	330	3 (0.9%)	10 (3.0%)	3 (0.9%)
	40-49	203	0	8 (3.9%)	5 (2.5%)
	50-59	268	0	6 (2.2%)	2 (0.7%)
	60-69	313	0	3 (1.0%)	0
	70-79	249	0	5 (2.0%)	2 (0.8%)
	80-	93	0	1 (1.0%)	0
					
	all	1930	7 (0.4%)	39 (2.0%)	15 (0.8%)
					
					
**Female**					
	10-19	165	3 (1.8%)	7 (4.2%)	1 (0.6%)
	20-29	476	9 (1.9%)	19 (4.0%)	3 (0.6%)
	30-39	480	9 (1.9%)	15 (3.1%)	4 (0.8%)
	40-49	289	4 (1.4%)	14 (4.8%)	2 (0.7%)
	50-59	413	5 (1.2%)	10 (2.4%)	3 (0.7%)
	60-69	401	1 (0.2%)	13 (3.2%)	1 (0.2%)
	70-79	375	0	7 (1.9%)	4 (1.1%)
	80-	143	0	3 (2.1%)	2 (1.4%)
					
	all	2763	31 (1.1%)	88 (3.2%)	20 (0.7%)
					

	Total	4693	38 (0.8%)	134 (2.9%)	35 (0.7%)

Table 3 shows the incidence of depression in patients who complained of headache. As demonstrated, 8.4% of patients who chiefly complained of headache had been diagnosed as depression while 1.7% of remained patients had been diagnosed as depression. The rate of depression was significantly high in patients with headache.

**Table 3:** Incidence of depression in patients who complained of headache or others

(A) Chief symptom		(B) Depression (B/A %)	
Hadache	418	35	(8.4%)
Others	4275	71	(1.7%)
			
Total	4693	106	(2.3%)

*, p < 0.001, when compared with others.

## Discussion

Out of 4693 patients, headache was the chief complaint in 418 patients (8.9%), indicating that diagnosis and treatment for headache is one of the most important clinical issues in outpatient units in Japan.

According to this study, approximately 50% or 60% if systemic infection was excluded out of patients with headache were diagnosed as primary headache such as TTH and migraine, and depression. We should therefore consider the three disorders, TTH, migraine, and depression, as major causes of headache in this particular clinical setting in Japan.

With regard to the prevalence of primary headache such as migraine and TTH, a number of reports have been published in many countries. Murtaza et al. [9] have demonstrated that out of 255 consecutive patients who presented to a headache clinic at a tertiary care hospital in Pakistan, migraine was the most common disorder (206 patients) followed by TTH (58 patients). According to a report by a Brazilian tertiary-care center [10], the prevalence of diagnosis in patients with headache were migraine (38%) and TTH (22%), respectively. Thus, in Pakistan and Brazil, migraine is the highest prevalence in patients with headache. However, in a large majority of countries, TTH is the highest prevalence in patients with headache [2].

A population-based survey in Japan (the Disen study) revealed the 1-year prevalence of migraine and TTH was 6.0% and 21.7%, respectively (4). A nationwide survey in Japan by Sakai et al. [3] have demonstrated that overall prevalence of migraine and TTH was 8.4% and 22.4%, respectively. The present findings obtained from an outpatient clinic-based study revealed migraine and TTH was diagnosed in 9.1% and 30.8% out of patients who complained of headache, respectively. These results suggest approximately 3 times higher prevalence of TTH than migraine not only in general population but also in outpatient units in Japan.

Jensen et al. [2] have demonstrated that the male: female ratio for migraine among adults varies from 1:2 to 1:3. A nationwide survey of migraine in the general population in Japan [3], the male: female ratio was 1: 3.6. The present study showed that male: female ratio for migraine was 1: 2.75, supporting the higher risk of migraine in female than male.

It has been demonstrated in the recent review that the male: female ratio for TTH is 4: 5, indicating that unlike for migraine, women are only slightly more affected than men [2]. In the present study, male: female ratio for TTH was 1: 1.6, furthermore supporting that women are only slightly more affected by TTH than men, when compared with migraine.

With regard to the relation of age and the prevalence of migraine, it has been shown that the highest prevalence of migraine was in women in their 30's, in whom one in five suffered migraine [1]. The present study similarly showed that migraine was observed higher in women in 20's and 30's. On the other hand, the rate of TTH peaks between the ages of 40 and 49 years in men and women. These results suggest that there are gender and age-dependent differences in the prevalence of between migraine and TTH.

Takeshima et al. [4] have demonstrated that 1-year prevalence of migraine was 6.0%, but less than 10% of migraine had consulted a physician in Japan. According to the report by Sakai et al. [3], nationwide survey of migraine was performed in Japan and demonstrated that the overall prevalence of migraine in the past year was 8.4% while doctor attendance rate was very low and approximately 70% with migraine had never consulted a physician for headache. The present study showed that migraine was diagnosed in only 0.8% out of all patients. Although there is a much difference in the prevalence of migraine in between the general population (6.0% and 8.4%) reported previously and outpatients (0.8%) examined in this study, the difference might be explained by the evidence that only a small part of migraine consult or seek medical service as described in above at least in Japan.

The present results also demonstrated that cerebrovascular disorders had been rarely observed in patients who complained of headache. The reason may come from the features in this hospital that a large majority of patients with severe headaches who might have cranial vascular diseases visited to our hospital by ambulance. Doctors in emergency room take care of the patients who come by ambulance. In this study, we analyzed the data of walk-in patients in this outpatient department. These are the reasons why cranial vascular diseases were rare in this study.

One may speculate whether diagnosis of depression was performed adequately in this study. We have recently published a paper on patients with prescription of selective serotonin reuptake inhibitor (SSRI)s, anti-depression drugs, in our clinic [5]. As described in the paper, SSRIs were prescribed to 126 (2.7%) patients out of all 4670 patients during 4 years. The percentage might be acceptable because major depression is seen by primary care physicians with a prevalence of approximately 5% in adult patients in western countries [11-13]. We would therefore suggest the ability of accurate diagnosis of depression would not be far from the estimated percentage of depression in our clinic.

The present study revealed that 8.4% out of patients who chiefly complained of headache were diagnosed as depression. On the other hand, 1.7% out of remained patients who chiefly complained of symptoms other than headache were depression, suggesting approximately 5 times higher prevalence of depression in patients with headache. Marlow et al. [14] have reported that 32% of patients with headache were depression whereas 12% patients without headache were depression, indicating 3-times higher prevalence of depression in patients with headache, supporting our present results. Chung et al. [15] have demonstrated that patients who presented with a chief complaint of headache in the outpatient family practice setting were found to have a high prevalence of depression. These evidence suggest that headache would be a marker of depression in the primary care setting also in Japan.

## Conclusion

As shown in this study, a majority of patients who visited to our outpatient unit because of headache were diagnosed as TTH and migraine, and depression. The epidemiological characteristics may help our daily clinical practice in outpatient units where primary care physicians are working in Japan.

## Competing interests

The authors declare that they have no competing interests.

## Authors' contributions

TO conceptualized, designed, collected and analyzed data and drafted the manuscript. ST, MO, ST and TN contributed to collection and analysis of data. All authors read and approved the final manuscript.
